# Protein Misfolding in Pregnancy: Current Insights, Potential Mechanisms, and Implications for the Pathogenesis of Preeclampsia

**DOI:** 10.3390/molecules29030610

**Published:** 2024-01-26

**Authors:** Bani Medegan Fagla, Irina Alexandra Buhimschi

**Affiliations:** Department of Obstetrics and Gynecology, College of Medicine, University of Illinois at Chicago, Chicago, IL 60612, USA; bmedegan@uic.edu

**Keywords:** protein misfolding, preeclampsia, amyloid-β, Alzheimer’s disease, tau, transthyretin

## Abstract

Protein misfolding disorders are a group of diseases characterized by supra-physiologic accumulation and aggregation of pathogenic proteoforms resulting from improper protein folding and/or insufficiency in clearance mechanisms. Although these processes have been historically linked to neurodegenerative disorders, such as Alzheimer’s disease, evidence linking protein misfolding to other pathologies continues to emerge. Indeed, the deposition of toxic protein aggregates in the form of oligomers or large amyloid fibrils has been linked to type 2 diabetes, various types of cancer, and, in more recent years, to preeclampsia, a life-threatening pregnancy-specific disorder. While extensive physiological mechanisms are in place to maintain proteostasis, processes, such as aging, genetic factors, or environmental stress in the form of hypoxia, nutrient deprivation or xenobiotic exposures can induce failure in these systems. As such, pregnancy, a natural physical state that already places the maternal body under significant physiological stress, creates an environment with a lower threshold for aberrant aggregation. In this review, we set out to discuss current evidence of protein misfolding in pregnancy and potential mechanisms supporting a key role for this process in preeclampsia pathogenesis. Improving our understanding of this emerging pathophysiological process in preeclampsia can lead to vital discoveries that can be harnessed to create better diagnoses and treatment modalities for the disorder.

## 1. Introduction

Proteostasis, which describes the process through which protein homeostasis is maintained in the body, is achieved through a collection of tightly regulated mechanisms and systems. These systems include regulation of protein synthesis at translational and post-translational levels, folding into structurally functional entities through a network of molecular chaperones, and degradation through autophagy or the ubiquitin–proteasome pathway [1]. Although these systems are tightly regulated, they must also be dynamic to adapt to fluctuating physiological conditions. Unfortunately, there are multitudes of conditions that are known to disrupt protein homeostasis, leading to aberrant polymerization and aggregation of inert or toxic higher-order protein species in tissues. It was even postulated that protein misfolding pathology could be an underlying factor in half of all human diseases [2,3]. Some of the well-studied disorders in which loss of proteostasis is known to contribute to pathogenesis include cystic fibrosis, diabetes, α-antitrypsin deficiency, cancer, and neurodegenerative disorders, like Alzheimer’s disease [3]. Protein misfolding is also known to be precipitated by aging or major physiological alterations and stress [4]. One such stress that involves drastic anatomic, metabolic, and cardiovascular changes is pregnancy.

Pregnancy has been described by Williams and David as a maternal stress test [5]. Indeed, pregnancy puts women through a series of rapid physiological changes that are necessary to accommodate the demands of gestation. Along with increased cardiac output and basal metabolic rate, increased glucose synthesis, increased insulin resistance, and hyperlipidemia, there is increased protein synthesis and turnover to accommodate the higher rate of tissue synthesis [6,7,8,9,10]. Consequently, it is not surprising that dysregulation in proteostasis may occur. In recent years, work by our group and others has led to the characterization of preeclampsia (PE), a life-threatening pregnancy-specific syndrome affecting 5–10% of all pregnancies worldwide, as a protein conformational disorder [11,12,13,14,15,16]. We, and others, postulated that PE could be a manifestation of the maternal body’s failure to accommodate the increased protein turnover, which leads to dysfunction in protein homeostasis mechanisms and the accumulation of potentially pathogenic misfolded proteins in the urine, serum, and placenta of women with PE. However, the cause of proteostasis dysfunction remains elusive. Hence, further exploration of protein misfolding characteristics in PE pathology and the identification of the mechanisms behind their existence is crucial to improving our understanding of PE pathophysiology and for the development of better diagnostic and treatment methods.

In this review, we will first summarize the current understanding of PE pathophysiology and existing gaps in knowledge. Second, we will discuss existing evidence of protein misfolding pathology in PE, including shared mechanistic processes between PE and other well-studied protein misfolding disorders. Third, we will explore advances in diagnostic approaches and potential treatment strategies based on the detection and targeting of misfolded proteins.

## 2. The Central Hypothesis of PE Pathophysiology and Current Gaps

While substantial progress has been made over the years to understand the pathophysiological mechanisms associated with PE, significant controversy remains. A chief challenge is that PE is not a single disease with a single causative pathway but rather a collection of highly heterogeneous clinical phenotypes with non-specific molecular and pathologic findings that often overlap with other pregnancy disorders [17,18]. Clinically, PE manifests as new-onset hypertension (systolic blood pressure ≥ 140 mmHg, diastolic blood pressure ≥ 90 mmHg) after 20 weeks of gestation with or without proteinuria [17]. This definition is a recent revision of the traditional clinical classification criteria for PE, which was based on the combination of hypertension and proteinuria [19,20]. The definition has been broadened to encompass cases without proteinuria but with evidence of maternal end-organ damage in the form of hemolysis, elevated liver enzymes and low platelet count syndrome (HELLP); renal damage; pulmonary edema; visual disturbances; persistent headache; stroke; and seizures (eclampsia) [19]. The current diagnostic and clinical guidelines distinguish between PE with and without severe features (sPE) based on the occurrence of any of these severe maternal symptoms or a systolic blood pressure over 160 mmHg and diastolic blood pressure above 90 mmHg [19]. As a result of this revision, an increased number of women with milder symptoms and a lower probability of adverse outcomes are now diagnosed with PE [21]. On the other hand, the clinical phenotype can also be subclassified based on the time of disease onset. PE arising before 34 weeks of gestation classifies as early-onset PE (EOPE) while PE arising after 34 weeks of gestation classifies as late-onset PE (LOPE). LOPE and EOPE have been shown to have distinct pathophysiological mechanisms and varying levels of disease severity [17]. While LOPE is associated with milder maternal disease, EOPE is often linked to severe maternal disease and superimposed fetal pathology manifesting as fetal growth restriction (FGR) [22]. The conditions and molecular pathways that drive one phenotype versus the other continue to be highly debated, fueling a lack of consensus on causative mechanisms.

It is widely accepted that the placenta, a transient but indispensable organ for pregnancy maintenance and fetal development, is the initial site where dysfunction arises. Indeed, the central hypothesis of PE pathogenesis supports a two-stage model with a defective invasion of extravillous trophoblasts cells (EVTs) and incomplete remodeling of spiral arteries at the maternal–fetal interface during placentation as the inciting factor for disease (Stage 1) [23,24,25]. In normal pregnancy, spiral arteries, which connect the placenta to the maternal blood supply, undergo extensive transformation. These blood vessels must widen to accommodate the increase in blood flow necessary to support a growing fetus [26]. This remodeling process has been shown to be mediated by the invasion of EVTs from the anchoring villi to the maternal decidua and deeper into the myometrium (interstitial trophoblasts). A subpopulation of the migrating EVTs also invades the wall and lumen of spiral arteries, where they replace endothelial cells (as intramural and endovascular trophoblasts, respectively) [26,27,28,29,30]. This process is compromised in PE, in which EVT invasion is inhibited and trophoblast-associated spiral artery remodeling fails. This failure of adaptation results in poor blood flow to the intervillous space, leading to placental hypoxia and ischemia. Stage 2 represents the development of the maternal syndrome characterized by generalized maternal vascular dysfunction [31,32,33,34,35]. Oxidative stress, endothelial damage, and soluble factors originating from the hypoxic placenta are among the pathogenic factors for Stage 2.

Despite well-established evidence of this pathophysiologic process occurring in PE, the shallow trophoblast invasion characterizing Stage 1 is not unique to PE. The failure of spiral artery transformation has also been described in preterm birth, fetal growth restriction (FGR), placental accreta spectrum disorders, and spontaneous miscarriage with varying degrees of changes and severity in placental phenotype [32,34,36,37,38,39]. Furthermore, although signs of abnormal placentation are commonly observed, they are not observed in all affected women, nor are they always sufficient to trigger clinical symptoms when present [29,40]. Intriguingly, the endothelial dysfunction resulting from placental malperfusion causes an imbalance in angiogenic markers, such as soluble fms-like tyrosine kinase-1 (sFlt-1) and soluble endoglin (sEng), which increase; and VEGF which decreases [41,42,43]. However, there is evidence that these changes in levels of angiogenic factors can be detected as early as 7 weeks of gestation, before the deep invasion of EVTs occurs, which suggests that vascular remodeling dysfunction may occur independently of trophoblast invasion [17,33,44,45]. For example, it has been established in human and animal studies that uterine natural killer (uNK) cells and macrophages play an important role in spiral artery remodeling in the decidua [46,47,48,49,50]. These immune-cell-mediated alterations, which include disruption of the vascular smooth muscle cell layer and endothelial cell loss, occur prior to EVT-associated events [45,46,47]. Aberrant complement system activation, impaired T-cell-mediated immune tolerance towards paternal antigens, and increased inflammation in the placenta are additional immune-system-related processes that have been documented in PE, supporting a maternal-driven immunological maladaptation etiology for the disorder as opposed to placental origins [51,52]. Finally, PE can present post-partum, when there is no longer a placenta, suggesting lingering systemic dysfunction that is not associated with the presence of the placenta. As the timeline and trigger of events endorsed in the current paradigm remain unclear, the need to identify additional pathophysiologic processes and markers that can clarify unsolved mechanisms is undeniable. Multiple hypotheses are still being entertained.

A multitude of environmental, genetic, fetal, and maternal risk factors that modulate PE risk and influence disease severity also exist. One striking example is the relationship between chronic kidney disease (CKD) and PE. On one hand, PE onset is associated with the development of acute end-organ damage involving vascular damage, glomerular endotheliosis, and chronic kidney damage, with a three–fifteen-fold increased relative risk of subsequent end-stage renal disease [53,54]. On the other hand, women who have pre-existing CKD are ten times more likely to develop PE compared to women without CKD [55,56]. Even more intriguing, women with a single kidney are three times more likely to develop PE than women with two kidneys, even in the setting of a normal glomerular filtration rate [57]. This epidemiological observation has also been demonstrated in vivo in a mouse model that exhibits a PE-like phenotype following unilateral nephrectomy [58].

**Figure 1:**
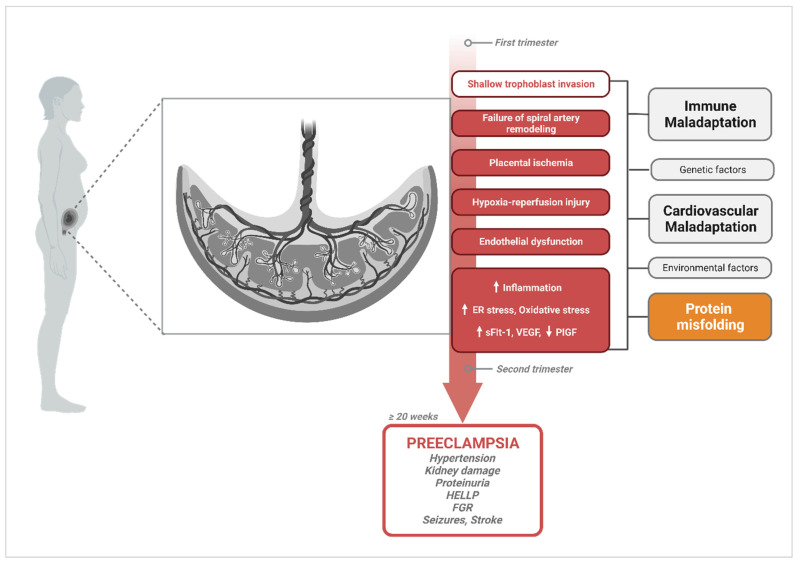
Diagram of pathophysiological processes described in the central hypothesis of PE pathogenesis, illustrating multifactorial etiologies that could trigger the widely accepted canonical pathway. Canonical mechanisms involved in PE pathogenesis and their progression to PE symptomatology are highlighted in red. Upward arrows indicate the pathological processes and vascular markers that increase in PE, while downward arrows represent markers that are known to decrease. A handful of other alternate or complementary etiological factors that have been proposed to contribute to these central mechanisms are shown in grey (immune maladaptation, genetic factors, cardiovascular maladaptation, environmental factors); and orange for protein misfolding, which this review will focus on. Created with BioRender.com, accessed on 18 December 2023.

The spectrum of potential placental, fetal, and maternal pathophysiological processes and risk factors involved in PE create considerable ambiguity in discriminating cause from effect. Hence, a multifactorial etiology of PE that involves abnormal placentation, immune maladaptation mechanisms, cardiovascular and renal dysfunction, as well as multisystemic disruptions in proteostasis cannot be dismissed (Figure 1). Findings suggesting that protein misfolding may be a leading contributor to PE pathology have increasingly emerged over the last decade, providing a new perspective on how we study disease mechanisms associated with PE.

## 3. Protein Misfolding in PE

### 3.1. General Protein Misfolding and Aggregation Mechanisms

Protein misfolding mechanisms and the protein conformational disorders (proteinopathies) that arise as a result have been extensively studied in the context of neurodegenerative disorders, such as AD, Parkinson’s, Huntington’s, and prion disease. More and more illnesses continue to be characterized as proteinopathies thanks to the advances made while studying these prototypical protein conformational disorders. It is well recognized that protein molecules can assume versatile secondary, tertiary, and quaternary structures or conformations based on their function and biological environment [1,59]. Polypeptide chains derived following mRNA translation must go through folding and assembly into well-defined native structures to assume the functional state of the protein. These processes occur primarily in the endoplasmic reticulum (ER), a subcellular compartment that serves many functions, including the acquisition of post-translational modifications, but can also take place in the cytoplasm [1,60]. Dysregulation of normal folding processes at this level, leading to improper folding and assembly into non-native proteoforms, can occur due to the failure of molecular chaperones, ER-associated protein complexes that facilitate proper protein folding, or due to disturbances in the physiologic environment that can trigger the abnormal polymerization of peptides [1,61]. Accumulation of abnormal protein conformations can also result from failure in protein degradation pathways, such as disrupted ubiquitin–proteasome signaling or ineffective cellular autophagy processes [62,63]. Lastly, as is the case in AD with Aβ, abnormal proteoforms can result from aberrant cleavage of native proteins (APP) [59]. The different conformational states that proteins can form and aggregate into exist on a spectrum: from the nascent peptide chain; to secondary β-sheet structures; to low molecular weight (LMW) oligomers; and, ultimately, into large protofibrils and fibril aggregates that form large extracellular deposits [59].

**Figure 2:**
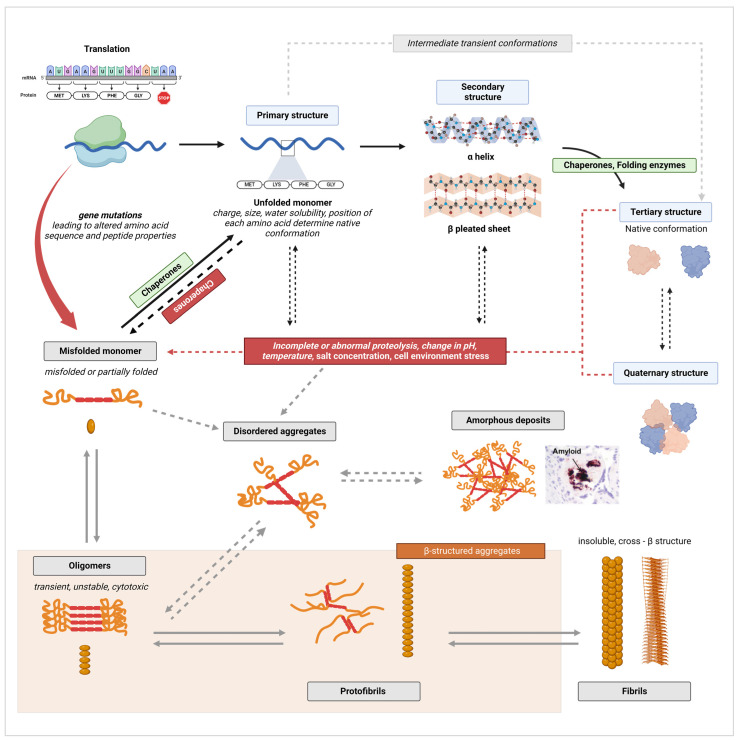
Illustrative diagram of native protein structure formation and amyloid fibril formation pathways. Canonical processes are indicated by full arrows, while alternate pathways are indicated by dashed arrows. Black arrows were used for processes of normal protein folding, while grey arrows were used to indicate fibrillation mechanisms. Created with BioRender.com, accessed on 20 January 2024.

The pathways of native protein structure formation and of amyloid fibril formation are contrasted in Figure 2. As established by decades of research in biochemistry and protein biology, the amino acid sequence of a peptide determines the unique functional conformation a protein will adopt [64,65]. Each unique protein sequence and each amino acid in the sequence carries intrinsic properties, such as size, charge, or water solubility (hydrophobicity or hydrophily), that drive the interatomic interactions responsible for shaping a protein into its optimal conformation [66]. A normal physiological environment is also fundamental for proper folding as native conformations must be thermodynamically stable under those conditions [65,66]. In ideal physiological conditions (which vary for each protein), an unfolded monomeric peptide (primary structure) will assume multiple potential conformational states (transient intermediates) that are accessible to the peptide until the most energetically favorable conformation is attained [59,65,66,67]. These intermediates include partially folded secondary structures, such as α helixes and β-pleated sheet structures, which form primarily through non-covalent interactions. These non-covalent bonds include electrostatic forces (ionic bonds); van der Waals interactions; and, most critically, intramolecular (both α helixes and β-sheet) and intermolecular hydrogen bonds (β-sheet only) [68,69,70]. In addition, the hydrophobic effect, which drives the burial of hydrophobic residues in the core of the protein and exposes hydrophilic residue to the outer surface, as well as covalent bonds, such as disulfide bonds, between side chains further stabilize polypeptides into a stable three-dimensional shape (tertiary structure and quaternary structure) [68,70,71]. Importantly, interactions with molecular chaperones, folding enzymes, degradation processes, and other cellular quality control mechanisms are critical for proper folding [2,66]. These mechanisms will be discussed in further detail in Section 5 of this review.

On the other hand, disturbances in the cellular environment, such as changes in pH, temperature, salt concentration, or aberrant proteolysis, can lead to the formation of misfolded peptides [72]. The aforementioned disturbances can cause the disruption of native bonds and aberrant non-covalent interactions, leading to misfolding (Figure 2). The thermodynamic stability of a protein’s native conformation can also be disrupted by protein concentrations that exceed a specific critical threshold [73]. In such conditions, the formation of aggregates becomes energetically more favorable than maintaining the native state [67,73]. Another way through which misfolded monomers can form is through aberrant or incomplete proteolysis of a native protein [66]. The failure of protein degradation systems can cause the fragmentation of a fully folded polypeptide into unfolded or partially folded fragments that are inherently prone to self-aggregation as these proteolytic fragments may expose segments of the protein hydrophobic core or backbone to abnormal molecular interactions [59,66]. Additionally, β-sheet-rich peptides and fragments in particular have a high propensity to aggregate as the pleated sheet structure facilitates the formation of interstrand hydrogen bonds favorable for polymerization [74,75]. Both changes in cellular milieu and proteolysis can simply denature the protein, leading to similar results [72]. While most proteins are unstable in their unfolded state, it is important to note that for some proteins, the unfolded state constitute their stable functional conformation [67]. Finally, genetic mutations that alter the amino acid sequence of a peptide, thus modifying the physicochemical properties of the original protein sequence, can initiate aggregation by making the formation of non-native interactions within and between peptide chains possible [74]. Mutations can also change the proteolytic cleavage sites of a polypeptide, resulting in the accumulation of aggregation-prone unfolded or partially folded fragments [76].

The first stage in the aggregation of unfolded, partially folded, or misfolded monomers is the formation of oligomers [66]. Oligomers are unstable polymers of at least four repeating units of a monomer, connected through weak and non-specific intermolecular interactions (Figure 2) [59,66]. As monomers continue to be added by self-association, oligomers transform from disorganized amorphous aggregates into more compact, structured, and stable aggregates known as protofilaments, which further self-assemble into protofibrils [59,66,77]. Two to eight protofibrils then intertwine on a lateral axis to form mature fibrils with β-strands running perpendicular to the fibril axis [59]. Extensive hydrogen bonding within and between β-sheets plays a critical role in stabilizing the highly ordered cross-β structure [72]. This self-assembly process occurs through nucleated polymerization [67]. The classical mechanism of nucleation is described as primary nucleation, in which the rapid self-propagation of monomers into larger fibrils is initiated once a nuclei, the smallest oligomeric species that can trigger self-growth and elongation, is formed [78,79,80,81]. For example, Paci et al. determined that oligomers of more than four units are required to initiate transthyretin (TTR) fibrillation [82]. There also exist secondary mechanisms, such as the fragmentation of existing fibrils into smaller aggregates that can act as new nuclei, and secondary nucleation, in which the surfaces of fibrils act as a catalyst to initiate the nucleation of new aggregates from monomers [78,79,80,81,83]. Importantly, disorganized aggregates may forego structural reorganization and form large amorphous amyloid deposits [66]. While this pathway of fibril formation is often presented as a hierarchical and linear process, proteins have significant plasticity in vivo and interconversion between different intermediates and conformational states is part of normal physiology [67,84,85].

**Figure 3:**
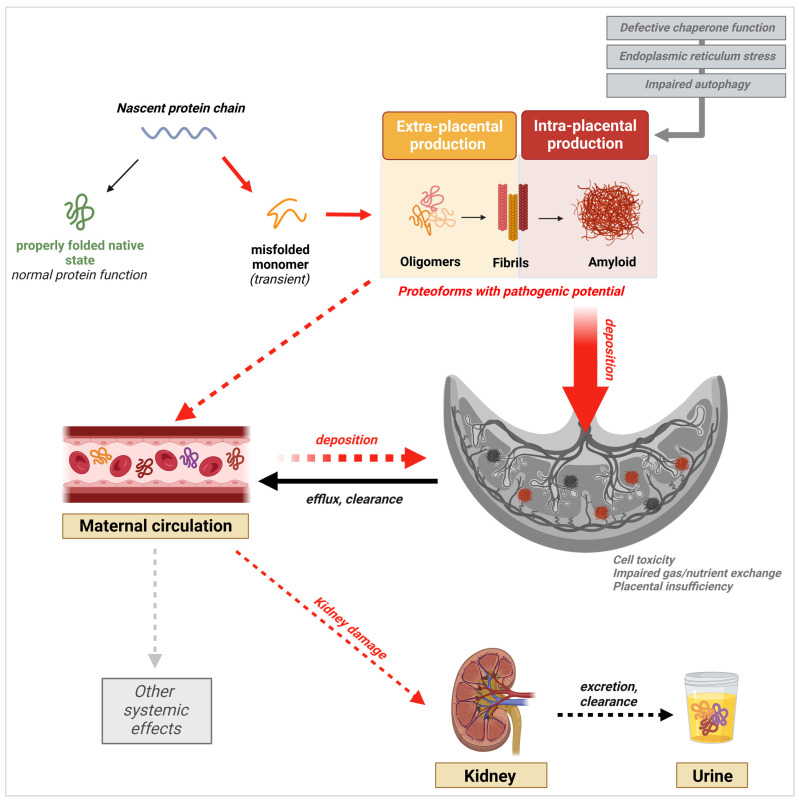
Schematic of the protein aggregation cascade and their potential multisystemic effects leading to or resulting from PE onset. Validated processes are shown by full arrows, while proposed potential mechanisms are indicated by dashed arrows. Created with BioRender.com, accessed on 20 January 2024.

In AD and other protein misfolding disorders, the presence of fibril deposits in tissue was thought to be the main pathogenic driver of disease. However, strong evidence supporting oligomers as the most toxic and pathogenic conformation has now emerged [86]. Oligomers have been shown to be highly cytotoxic to neuronal cells and to disrupt cell membrane integrity, calcium homeostasis, and essential cellular processes and signaling pathways [86,87]. This knowledge has triggered a similar shift in our understanding of other proteinopathies, including PE. Indeed, the latest research findings demonstrate the presence and potential pathogenicity of prefibrillar species of diverse proteins in PE. Nevertheless, the evidence also shows that fibrils and large amyloid plaques are present in the placenta, as they are in the brain of AD patients, supporting a role for both species in PE pathophysiology, albeit through different mechanisms (Figure 3) [87]. In contrast to oligomers, fibrils have been shown to be relatively inert in vitro and in vivo [86,88]. However, they may still contribute to disease mechanisms through loss of protein function in the amyloid state, or through the generation of toxic prefibrillar species during their formation and during fragmentation [67,88].

### 3.2. Excretion of Misfolded Proteins in the Urine

Initial clues that protein misfolding pathology occurs in PE came to light when our group identified the presence of supramolecular aggregates of SERPINA1 (alpha-1 antitrypsin, A1AT) in the urine of women with sPE, along with conformational aggregates (including oligomers) of the protein in the placenta [13]. Since proteinuria is a major clinical manifestation of PE, we further verified the presence of misfolded proteins in the urine of women with sPE compared to gestational age-matched controls using Congo red, a dye that exhibits high binding affinity for amyloid proteins with a β-sheet conformation and is the gold standard for post-mortem amyloid plaque detection in AD [12,89]. We reported that women with sPE exhibited urine congophilia, a property that confirms the presence of protein species made of the large polypeptide chains that are characteristic of misfolded proteins [12]. This congophilic property was highly specific to PE compared to other hypertensive disorders of pregnancy (gestational and chronic hypertension) and Congo red retention (CRR, a measure of congophilia) was associated with disease severity [12]. Using conformation-specific antibodies, we confirmed the presence of prefibrillar oligomers and protofibrils but not fibrils in sPE urine; although, not all sPE urine samples with congophilia displayed immunoreactivity to these conformational species [12]. Importantly, using a mass-spectrometry-based approach and Western Blot analysis, we identified the presence of albumin, SERPINA1, ceruloplasmin, interferon-inducible protein 6–16 (IFI6), IgG k-free light chain, and amyloid precursor protein (APP) proteoforms as part of the urine amyloid precipitates from sPE [12]. An independent study by McCarthy et al. also confirmed urine congophilia as a characteristic of PE in a cohort that included non-pregnant women with systemic lupus erythematosus (SLE), pregnant women with gestational hypertension or chronic hypertension, and pregnant women with chronic kidney disease (CKD) with and without superimposed PE [90]. The study found that urine congophilia was also present in women with CKD, independently of the occurrence of PE, and in comparable levels to women with PE alone. Furthermore, non-pregnant women with SLE-associated nephritis also had an elevation in CRR compared to non-pregnant controls. These results are supportive of a systemic or extra-placental origin of disruption in proteostasis. The existence of urine congophilia in women with CKD or SLE nephritis also challenges the specificity of the clinical finding to PE. In pregnant women with pre-existing kidney disease who go on to develop PE, the culprit of amyloid protein aggregation would be indistinguishable. It is also possible that the presence of amyloid preceding PE onset contributes to the increased incidence of PE in women with kidney disease. A third study evaluated whether urine congophilia was present in a cohort of Indian women presenting with mild or severe PE [91]. In this cohort, pregnant women with common comorbidities, such as chronic hypertension, diabetes, and cardiovascular disease, were excluded. Compared to normotensive patients, women with mild PE and sPE had a 1.6-fold and 2.2-fold increase in CRR respectively. In the study, Nagarajappa and co-authors also compared CRR between distinct clinical sub-phenotypes of the disease, including LOPE, EOPE, eclampsia, and PE with fetal pathology (FGR or intrauterine fetal demise). All subclasses had significantly higher percentages of CRR compared to controls. Although the urine congophilia phenotype in PE patients provides helpful insight into the existence of protein misfolding pathology in PE, it is still unclear where the abnormal proteoforms detected in urine originate from.

To evade this challenge, using approaches that allow for the detection and identification of specific proteins may have more potential in discerning the cause of the pathophysiologic protein aggregation process. For example, mass-spectrometry-based approaches helped identify and confirm the presence of SERPINA1 peptides in PE urine, including fragments located in C-terminal regions of the protein known to be highly amyloidogenic [13,92,93]. Immunodetection techniques, such as Western Blot analysis and dot blot immunoassay profiling, have been used to confirm the presence of oligomeric proteoforms of SERPINA1 and Aβ in sPE urine [13,94]. In both studies, conformational-dependent antibodies were used, allowing for a closer look at the secondary structure of the proteins identified. Improving our understanding of the urine-specific PE misfoldome is crucial for the development of detection methods with high sensitivity and specificity that can be used for clinical diagnosis. More evidence supporting the role of misfolded proteins in PE pathogenesis came with the detection of misfolded proteins in the serum of women with PE.

### 3.3. Presence of Misfolded Proteins in the Maternal Circulation

Kalkunte et al. demonstrated the presence of aggregated proteoforms of transthyretin (TTR) in the serum of women with PE, providing additional supporting evidence for the protein misfolding hypothesis of PE pathophysiology [11]. They also showed that the intraperitoneal injection of PE serum or TTR directly extracted from PE serum induced a PE-like syndrome characterized by hypertension, glomerular endotheliosis, fetal growth restriction, proteinuria, and increased sFlt-1 and sEng in IL-10^-/-^ mice compared to mice injected with serum from normal pregnancies [11]. The PE-like phenotype was rescued when PE serum was co-administered with exogenous TTR in its native form or when TTR was depleted from PE serum [11]. These findings highly suggest that the presence of these abnormal TTR aggregates is causative of PE and not a result of any preceding dysfunction. Using a cell-based in vitro model for the detection of protein aggregates, Cheng et al. also reported the presence of Amyloid-β in addition to TTR in the serum of women with EOPE and LOPE [15]. Using the same technique, Jash et al. confirmed that aggregated forms of the cis stereo-isoform of P-tau231 (phosphorylated at threonine residue 231), a protein isoform linked to pathogenicity in AD, are present in the serum of women with EOPE and LOPE but not the gestational-age-matched controls. The presence of multiple proteins that have been proven to be pathogenic in AD in PE reinforces the idea that the aggregation and accumulation of these proteoforms in PE serum is not harmless. In their study, Cheng et al. also tested the presence of SERPINA1 aggregates in PE serum but found it to be undetectable; although, our group had identified SERPINA1 in the urine and placenta of sPE patients [13]. This implies that different species may have tissue-specific patterns of deposition. Questions about the origin of the protein aggregates that could explain why certain proteins are present in some tissues but not others remain to be answered. The detection of misfolded proteins in urine and serum suggests that pathology may occur at a systemic level; however, their presence in the placenta during pregnancy could contribute directly to placental dysfunction and subsequent pathology secondary to tissue damage.

### 3.4. Placental Deposition of Extracellular Protein Aggregates

Our group has previously reported the presence of amyloid-like deposits of SERPINA1, APP, and Aβ in the placentas of women with sPE [12,13]. Detection of SERPINA1 aggregates using ATZ11, an antibody specific for oligomeric conformations of the protein, revealed increased placental deposition of misfolded SERPINA1 complexes in the stroma, endothelium, and fetal blood vessels of placentas from sPE pregnancies compared to gestational-age-matched controls [13]. Furthermore, these deposits were predominantly localized in the endothelium. Given the central role endothelial damage plays in PE pathophysiology, and the well-documented toxicity of oligomeric protein conformations, the perivascular localization of these deposits could be an indication that they contribute to endothelial dysfunction [86,95]. Another possibility, proposed by Starodubtseva et al., is that the placental deposition of SERPINA1 could be a protective mechanism against PE-induced hypoxia [92]. However, the researchers based their conclusion on the immunodetection of total SERPINA1 and not misfolded conformations of the protein. Whether misfolded forms of SERPINA1 participate in any of the characteristic processes associated with PE remains to be elucidated.

Abnormal proteolytic cleavage of APP has been established as the mechanism for Aβ formation, aggregation, and accumulation in the AD brain [59,87,96]. Similarly, hyperphosphorylation of tau, leading to the aberrant accumulation of pathogenic conformations of the protein, occurs [96,97]. Together, the deposition of toxic oligomers and large extracellular fibrillary plaques of Aβ with neurofibrillary tangles made of hyperphosphorylated tau are the two principal hallmarks of AD pathology. Along with the detection of misfolded SERPINA1, our group identified the presence of APP aggregates in the urine and placentas of women with sPE. Immunostaining of the placental tissue with ALZ90, an antibody specific for the Aβ fragment of APP, showed a positive signal for amyloid-like plaques of Aβ within areas of fibrinoid deposition [12]. This phenotype was accompanied by significant upregulation of β-secretases, the enzymes that are responsible for the abnormal cleavage of APP into Aβ, in the trophoblast layer of placental villi. These observations led us to propose that the placental or systemic clearance of misfolded proteins might be deficient in PE, leading to a supraphysiologic load of these species in the urine. Interestingly, Jash et al. not only reported the presence of misfolded cis P-tau in the serum but also in placentas from LOPE and EOPE pregnancies [98]. The cis P-tau deposits localized to the trophoblast layer in the villous structure and co-localized strongly with ProteoStat signal, a rotor dye specific for aggregated proteins [98]. Given the crucial role trophoblast cells play in vascular remodeling, hormone synthesis, and maternal-fetal gas and nutrient transport, the localization of aggregated cis P-tau at the trophoblast cell layer could directly impair placental function and induce placental insufficiency. Indeed, in vitro evaluation of the effect of the proteoforms showed that cis P-tau aggregates disrupted cell-to-cell communication between extravillous trophoblasts cells and endothelial cells, an essential process in spiral artery remodeling [98]. The research team was also able to induce the expression of aggregated cis P-tau in primary human trophoblast cells (PHTs) following a hypoxia-reperfusion protocol, highlighting that placental cells can produce abnormal proteoforms in response to hypoxia [98]. This would mean that misfolded proteins are synthesized as a result of pre-existing placental insufficiency and may not precede placental dysfunction.

Following the detection of aggregated proteoforms of TTR in PE serum, extensive studies have been conducted to understand their role in PE-associated placental pathology. Studies by Kalkunte et al. and Tong et al. revealed TTR deposition in the villous stroma and extravillous trophoblast cell layer in the human placental villi section [11,99]. The regions with positive TTR staining matched Thioflavin-S-positive regions, confirming that the TTR placental deposits in question were aggregated forms of the protein [11]. Previous work by Kalkunte et al. demonstrated that PE serum containing TTR aggregates also caused the disruption of endovascular activity and crosstalk between endothelial cells and trophoblasts. In this work, the investigators further show that the supplementation of PE serum with unaggregated TTR monomers reversed the PE serum-induced disruption of endothelial function in vitro and in vivo, offering a potential mechanism of action for the role of misfolded TTR in PE pathophysiology [11]. Going one step further, Tong et al. investigated the placental production of TTR [99]. They found that total TTR protein levels were significantly increased in placentas from preeclamptic patients compared to placentas from normotensive patients [99]. Additionally, in preeclamptic tissue, the protein was predominantly localized within the syncytiotrophoblast cell layer. Interestingly, placental expression of TTR mRNA did not differ between the PE and control group, suggesting that the increase in protein levels associated with PE is not due to an intrinsic increase in gene expression [99]. Notably, this study demonstrates for the first time that monomeric and aggregated forms of TTR are transported out of the placenta in nano-vesicles, with significantly higher protein levels in nano-vesicles from preeclamptic placentas compared to nano-vesicles from normotensive placentas [99]. Moreover, the PE-derived nanoparticles transported more aggregated protein compared to the controls [99]. These results suggest that in PE, misfolded proteins like TTR may be present in excess, in which case they are targeted for active efflux into the serum and transported out of the placenta in nano-vesicles. This proposed mechanism could account for the increased fraction of aggregated TTR in PE serum and explain how systemic effects may occur as a result of PE-associated misfolding. Nevertheless, this does not eliminate the possibility that a portion of circulating TTR abnormal proteoforms can still originate from other tissues.

Although these latest research findings shed light on the placental metabolism of aggregated proteins, like Aβ, cis P-tau, and TTR, and offer a potential source for the aggregated proteoforms detected in serum, the conditions in which specific proteins are formed are still unclear. It also appears that pathogenic forms from different proteins may impact placental function at the same time, with some being more predominant than others. A deeper look into the function and characteristics of each of these proteins may help explain their presence and effects in PE.

## 4. PE-Associated Misfoldome

A full list of the amyloidogenic proteins that have been investigated in PE is presented in Table 1. The following discussion will cover the function of six predominant protein species that have been confirmed across several of the eight major publications that explored the role of protein misfolding in PE. These proteins are SERPINA1 and its highly amyloidogenic FVFLM fragment, Aβ and its precursor APP, cis P-tau, and transthyretin.

### 4.1. SERPINA1/α-1-Antitrypsin (A1AT)

Serine protease inhibitors, also known as serpins, are a superfamily of proteins involved in the regulation of proteolytic enzymes throughout the body [100]. Serpins, especially SERPINA1, have been involved in a multitude of pathogenic processes, leading to a group of disorders recognized as serpinopathies. Notably, genetic mutations in SERPINA1 lead to severe and uncontrolled lung damage due to failure in the inhibition of neutrophil elastase, an abundant protease in the lung. In the liver, the main site of the synthesis of SERPINA1, defective SERPINA1 molecules accumulate and aggregate into higher-order polymers that induce fibrosis and cirrhosis [101]. Similarly, mutations in neuroserpin, a neurotropic serpin, cause familial encephalopathy with neuroserpin inclusion bodies (FENIB), a type of hereditary dementia characterized by the accumulation of neuroserpin polymers in neurons [100,102]. This tendency of serpins to polymerize is due to a native structure composed of three β-sheets, a property that is well-tailored to their proteolytic function [103,104]. As such, they are susceptible to uncontrolled β-interlinkages that lead to the formation of oligomers with toxic properties through polymerization [95,105,106,107]. Therefore, serpinopathies model the same mechanisms that result in conformational disorders.

SERPINA1 is also an acute-phase reactant that is upregulated during inflammation, infection, and pregnancy [101]. Thus, a role for the protein in PE pathophysiology is not unlikely. For example, the protein has well-documented anti-inflammatory properties that are independent of its proteolytic function [108]. The increased placental and systemic inflammation, which are characteristic pathophysiological processes in PE, could potentially drive increased SERPINA1 expression as a protective mechanism [92,109]. On the other hand, Twina et al. showed that sPE is associated with lower circulating levels of SERPINA1, in which case dysregulation in SERPINA1 levels could be the cause for increased inflammation [110]. It has also been proposed that SERPINA1 protects against the development of PE through the suppression of oxidative stress both in vitro and in vivo [111,112]. However, excessive expression of SERPINA1 in the placenta may favor aberrant oligomerization, inflicting further damage to tissue, as occurs in liver tissue in cases of A1AT deficiency. Others have argued that SERPINA1 increases the risk of PE by limiting trophoblast invasion into the maternal decidua via inhibition of matrix metalloproteinases, like MMP2, MMP9, or ADAMS12, which play a leading role in the process [92,113]. This hypothesis is supported by an inverse correlation between increased circulating SERPINA1 levels and decreased ADAMS12 expression [92,113,114]. Yet, Yoshida et al. demonstrated that endogenous SERPINA1 (A1AT) stimulates HTRA1-mediated extravillous trophoblast invasion by promoting ER stress, a mechanism independent of MMP2 and MMP9 levels [115]. Hence, decreased availability of functional A1AT molecules in the context of aberrant excessive polymerization has the potential to cause defective trophoblast invasion, leading to PE.

An important question is whether any of the known mutations that induce SERPINA1 oligomerization and deficiency exist in PE. Nagarajappa et al. conducted a cross-sectional study of 200 preeclamptic women to characterize the frequency of PiS and PiZ mutations, the two dominant mutations in the SERPINA1 gene that cause A1AT deficiency [116]. Results showed that these two variants were non-existent in the study cohort; however, the presence of other unidentified pathogenic mutations cannot be excluded [117]. The origin of SERPINA1 dysregulation and misfolding in PE is still unclear. Supposing the dysregulation results from established pathology as a compensatory mechanism instead of triggering it, there is still a high potential for a double-whammy effect due to the protein’s propensity to aggregate into toxic oligomers. The presence of highly amyloidogenic SERPINA1 fragments, such as the FVFLM peptide in the urine of women with PE, supports that excessive synthesis and release of toxic SERPINA1 proteoforms in the circulation either by the placenta or other organs occurs. The resulting urinary excretion of these peptides could be a clearance mechanism that helps remove these toxic species from the body [13,93]. Interestingly, molecular dynamics simulation models have demonstrated the capacity of the FVFLM peptide to bind to the Aβ^16–20^ fragment KLVFF, consequently inhibiting a binding sequence that is required for Aβ fibrillation [106]. This interaction offers a potential explanation as to why both SERPINA1 and Aβ aggregates are detectable in PE; however, more work needs to be conducted to verify the potential of SERPINA1 to inhibit Aβ aggregation in vivo. Additionally, the interaction between SERPINA1 and Aβ is not unique. Notably, SERPINA1 has also been shown to regulate the expression of TTR, another amyloidogenic protein relevant to PE [118]. Specifically, loss of SERPINA1 expression, via knockdown, increases TTR gene and protein expression in liver cells. Furthermore, SERPINA1 knockdown in mice causes a significant increase in circulating plasma levels of TTR and increased TTR deposition in multiple organs [118]. Although these findings were not obtained in the context of pregnancy, they show that a similar relationship between SERPINA1 and TTR could occur in the context of PE.

### 4.2. Aβ and Its Precursor APP

Despite ample evidence of the presence of misfolded Aβ and APP in PE serum, placenta, and urine, mechanistic studies on their effects in PE are sparse [12,15,94]. However, the aggregation of Aβ, which is a pathognomonic feature of AD pathology, is an uncanny similarity that can be exploited to better understand the mechanisms at play in PE. Thus, based on knowledge gathered from studies of Aβ pathology in AD, we are able to make inferences on what processes involving Aβ aggregation might be at play in PE. In AD, the amyloid cascade hypothesis is the classic view of pathogenesis [96,119]. According to this hypothesis, the deposition of Aβ aggregates drives neurotoxicity and the onset of dementia in AD [119]. Aβ monomers are a cleavage product resulting from the non-canonical processing of APP by β- and γ- secretases enzymes (BACE1, BACE2, PS1, PS2) instead of α-secretases (ADAM10) [120,121]. Importantly, our previous results show that BACE 1 and PS1 were highly expressed by placental trophoblast cells [12]. This implies that placental cells have the capacity to cleave APP into Aβ monomers that can further aggregate into pathogenic species. Aβ oligomers, and not monomers or fibrils, are now widely accepted as the pathogenic form that drives neuronal dysfunction. Their presence in the brain has been linked to neuronal death, disruptions in calcium homeostasis, the inhibition of plasticity, disruption of cell membrane integrity, neuroinflammation, oxidative stress, and aberrant tau phosphorylation [86]. These pathophysiological processes, which are traditionally attributed to hypoxia and ischemia, have also been linked to placental dysfunction in PE [122]. Hence, it is possible that once formed or deposited in placental tissue, oligomeric species of Aβ drive similar detrimental effects on placental cells. Evidence presented by Valtanen et al. showing that protein conformations analogous to those formed by Aβ oligomers are present in PE urine and correlate with disease severity supports the notion that, regardless of source, these aberrant proteins may worsen pre-existing hypoxia-induced pathological processes [94].

APP is a large multimodal protein involved in transcriptional regulation, cell survival, and synaptic plasticity in the brain; however, little is known about its physiological function in pregnancy [120]. APP is abundantly expressed in the placenta, and, more specifically, villous trophoblasts [106,123]. To date, a single study has investigated, in depth, the role of APP in placental physiology [123]. The authors show that APP overexpression induces apoptosis and reduces cell migration, invasion, and syncytialization. Importantly, mass spectrometry after APP induction highlighted the upregulation of proteins involved in protein folding processes [123]. It is unknown how these effects of APP on placental function are affected in the context of protein aggregation.

### 4.3. Tau

Alongside Aβ, tau aggregation is a major driver and hallmark of AD symptomatology. Tau is a microtubule-associated protein that aids in the assembly and stabilization of microtubule filaments. In the nervous system, these functions are critical for neuronal maturation and axonal transport [124]. Critically, the protein’s function is regulated via multiple post-translational modifications, the most common of which is phosphorylation at various residues [125]. Phosphorylation of tau induces its dissociation from microtubules, causing alterations in cytoarchitecture and poor microtubule stability [124,125]. In AD, and other tau-associated proteinopathies, hyperphosphorylation of tau at specific sites is known to induce neurotoxic effects, including self-aggregation into pathogenic helical filaments that make up neurofibrillary tangles and toxic intracellular inclusions [125]. As aforementioned, Jash et al. discovered a pathogenic role of cis P-Tai231, representing a major leap in our understanding of mechanisms driving protein misfolding in PE [98]. They established that the presence of aggregated cis P-tau231 causes disorganization of β-tubulin in the cytoplasm of trophoblast cells, similarly to its effect on neurons. Providing further clarification on how cis P-tau231 aggregates accumulate in the placenta, they show that Pin-1, an isomerase whose function is to convert cis P-tau231 to its non-pathogenic trans isomer, is inactivated in hypoxic conditions, thus driving the accumulation of cis P-tau231. This mechanism has also been documented in AD and other neurodegenerative diseases [98]. These studies further support the notion that pregnancy, which occurs in the earlier years of life, can mimic pathology typically associated with aging, emphasizing the state of pregnancy as a natural stress test.

### 4.4. TTR

TTR is an essential carrier protein that transports thyroxine and retinol-binding protein (RBP) bound to retinol (Vitamin A) [126]. It is primarily synthesized by the liver, choroid plexus, and retina before being secreted into the circulation and cerebrospinal fluid (CSF) [126]. Studies have shown that placental villous trophoblasts also synthesize TTR and that, in PE, an increased amount of placental extracellular vesicles carrying aggregated TTR as a cargo were released into maternal circulation [99,127]. Recent work by Cheng et al. also characterized the temporal expression of TTR during pregnancy [128]. Authors show that serum TTR levels decrease as gestation progresses, reaching a low point at around 13 days of gestation (mice) or 13 weeks of pregnancy (humans) while normal circulating levels are restored at term and post-partum [105]. Importantly, the decrease in circulating levels of TTR was associated with decreased liver and placental protein and mRNA expression, indicating that tight systemic regulation of TTR is important for normal pregnancy [105]. Indeed, in transgenic mice that overexpress human TTR, fetal growth and survival were significantly impaired [128]. Moreover, in IL-10^−/−^ mice, a mouse model that exhibits a pro-inflammatory phenotype, TTR levels were decreased compared to WT mice and WT levels were restored upon the administration of exogenous IL-10, suggesting that inflammation significantly inhibits TTR synthesis [105]. This work highlights a key role of TTR in pregnancy and indicates that inflammation, a dominant pathophysiological process in PE, can negatively disrupt the bioavailability of TTR. As the authors propose, it is possible that the downregulation of TTR, especially in the context of placental inflammation, could be a protective mechanism to control aberrant TTR misfolding given the highly amyloidogenic nature of the protein [105].

One essential function of TTR that could also contribute to PE pathophysiology is its role in angiogenesis and maintaining blood–brain barrier integrity in AD [129,130]. Nunes et al. showed that V30M, an amyloidogenic hereditary variant of TTR, causes the downregulation of pro-angiogenic factor expression, induces apoptosis, and reduces cell migration in endothelial cells [130]. Analogous effects of TTR aggregates on placental vasculature, including the impairment of capillary tube formation and induction of the anti-angiogenic factors sFlt-1 and sEng, have been described by Kalkunte and al [11]. We can also learn more about the potential effects of TTR misfolding from other TTR-associated proteinopathies. TTR mutations like V30M or age-driven misfolding and aggregation of wild-type TTR are involved in familial amyloid polyneuropathy (FAP) and senile systemic amyloidosis (SSR), respectively [129]. Both disorders are characterized by the deposition of TTR fibrils in the peripheral nervous system in the case of FAP and, primarily, in cardiac tissue for SSR. The pathophysiology of these disorders mimics that of other proteinopathies, including the characteristic tissue and cellular damage inflicted by toxic oligomeric intermediates [131]. But, in these disorders, the deposition of amyloid fibrils causes the harmful structural distortion of myelinated axons (FAP) and myocardium stiffening and fibrosis (SSR) [132,133]. Investigation of the structural impact the deposition of fibrils and large space-occupying amyloid lesions can have on the placenta has been lagging compared to research focused on effects that can be largely attributed to oligomers [11,99,125]. As amyloid fibrils can restrict cardiac mechanical function in SSR, we speculate that their presence in the placenta can reduce the available area for maternal–fetal exchange, thus, further weakening placental efficiency.

Studies investigating the role of TTR dysregulation and aggregation in driving PE-associated dysfunction also lay out two potential mechanisms contributing to the production and deposition of protein aggregates in the PE placenta: increased ER stress and impaired autophagy [134]. Studies looking into mechanisms of Aβ aggregation highlight a third major mechanism: dysregulation of chaperone proteins [135,136]. Together these studies paint a clear picture of how protein aggregation may come about in PE.

## 5. Driving Mechanisms of Protein Misfolding in PE

### 5.1. ER Stress and Hyperactivation of the Unfolded Protein Response (UPR)

ER stress is a well-defined feature of PE [23,35,60]. It is also a leading pathophysiologic process in protein conformational disorders [137]. The ER is a focal site of protein synthesis and maturation. Following mRNA translation by ribosomes in the cytoplasm, peptides are translocated to the ER lumen, where they undergo necessary post-translational modifications, folding, and assembly into their functional conformation. To carry out these functions properly, the microenvironment in the ER lumen must maintain a high concentration of Ca^2+^ and a high oxidative capacity [60]. In PE, chronic low oxygen tension in the placenta, as a result of impaired uteroplacental perfusion, disrupts the delicate balance of the ER milieu, therefore inhibiting proper function [60,109]. This state of ER stress leads to the accumulation of unfolded proteins, which are more prone to misfold and aggregate, as the ER functional capacity is diminished [138]. Common ER stress markers, such as ATF6, GRP78 (BiP/HSPA5), Ire1α, CHOP, PERK, and eIF2α, are upregulated in the PE placenta [109,134,138,139,140,141,142,143]. In fact, these markers are part of a set of regulatory pathways that are aimed at restoring proteostasis in the setting of ER stress. Together they constitute the UPR and function to slow down protein translation, ramp up the protein folding machinery, or induce apoptosis in case all attempts at containing damages fail [138,144].

While UPR activation normally acts as a protective mechanism, an excessive and prolonged UPR, as would be the case during chronic tissue hypoxia, is self-damaging. Ultimately, cellular response mechanisms can be overwhelmed, leading to a collapse of protective systems and dooming cells for apoptosis. Furthermore, misfolded proteins themselves aggravate ER stress. This detrimental self-seeding loop has been observed in the majority of protein-conformational diseases, including PE [122,134,145,146]. Using aggregated TTR as a model, Cheng et al. showed the two sides of the coin [134]. In PHTs, exposure to brefeldin A, a known inducer of ER stress, increased the deposition of TTR aggregates. Exposure to hypoxic stress had the same effect in PHTs and in TCL-1 cells. On the other hand, the accumulation of TTR aggregates coincided with the over-activation of UPR markers [142]. The aforementioned evidence supports that hyperactivation of the UPR could lead to increased protein aggregation in the placenta.

Given the important role ER dysfunction plays in the pathogenesis of protein conformational disorders, strategies aimed at inhibiting ER stress and UPR pathways are currently being investigated as a therapeutic approach to AD treatment [147]. Perhaps similar methods can be examined to minimize hypoxia-associated placental dysfunction in the context of PE.

### 5.2. Failure of the Protein Folding Machinery: The Role of Chaperones

Transcriptional upregulation of chaperones and folding enzymes is one of the compensatory mechanisms that are enacted by the UPR response. Under normal physiologic conditions, chaperones are molecules that aid other proteins in acquiring their natural conformation. They also have the capacity to recognize and bind to misfolded proteins, hence inhibiting or undoing their aggregation; and the capacity to unfold and refold improperly folded polypeptides, or tag them for degradation [61]. ER stress is not the only inducer of chaperone activity. For example, heat shock proteins (HSPs) are a ubiquitously expressed family of chaperones that are upregulated in response to a variety of stressors that include heat, oxidative stress, sterile inflammation, and viral infection [148,149]. Members of the HSP70 family, in particular, are notoriously involved in the regulation of decidual and placental cell function from placentation to the end of pregnancy [150]. Their upregulation in PE not only serves as a marker of UPR activation but also indicates that their overactivation could be driving pathological processes. If misfolded proteins are present in excess, increased expression of HSP70 isoforms like GRP78 is necessary for re-folding and disaggregating already tangled proteins. On the other hand, when bound to misfolded proteins, they are no longer available to perform other functions that are critical to maintaining cell integrity. This has been proposed as another possible mechanism driving the toxicity of protein aggregates like polyglutamine, an amyloidogenic protein involved in Huntington’s disease pathogenesis [151].

In PE-associated misfolding, pregnancy zone protein (PZP) has also emerged as an important player. In contrast with GRP78, which is an ER-associated intracellular chaperone, PZP has recently been characterized as an extracellular chaperone that can inhibit the aggregation of Aβ [135]. PZP is a dimeric homolog of the alpha-2-macroglobulin (α2M) that is found in high concentrations in the serum of pregnant women while levels are characteristically low in non-pregnant women and men [152,153,154]. With a 71% degree of homology between the two protein sequences, it has been proposed that they may perform similar functions [152,155]. Notably, α2M is a circulating protease inhibitor that has a well-established association with amyloid formation in AD [156]. Additionally, α2M co-localizes with amyloid plaques in the AD brain and has been shown to bind with Aβ in vitro [157,158,159]. Furthermore, α2M has the capacity to prevent the formation of Aβ fibrils and take part in the degradation and clearance of Aβ [160,161]. PZP also co-localizes with senile plaques in AD and circulating levels of the protein are increased in AD, even before the onset of clinical symptoms, warranting further investigation of its molecular role in mechanisms of protein aggregation [162]. Cater and al. later demonstrated that PZP, purified from human pregnancy plasma, stably binds to Aβ monomers and LMW oligomers in vitro. In an aggregation assay, PZP also inhibited Aβ fibrillation more effectively than α2M [135]. On the other hand, in situ, co-immunodetection of PZP in the placenta of PE patients shows no interaction between PZP and extracellular Aβ deposits. These results suggest that PZP may participate in the stabilization of unstable protein conformations before higher-order fibrils are formed. It is possible that the upregulation of PZP in normal pregnancy serves as an adaptive mechanism to minimize the formation of protein aggregates. Accumulating evidence shows decreased levels of PZP in the serum and placentas of women with PE, including severe phenotypes of the syndrome accompanied by FGR or HELLP [163,164,165]. This could constitute a failure of normal adaptation, leading to a diminished placental capacity to prevent the formation of aggregates.

Plasminogen activator inhibitor type 2 (PAI-2), another extracellular chaperone, has been linked to PE-associated protein aggregation [136]. Similarly to PZP, PAI-2, also known as SERPIN2B, is normally upregulated in pregnancy [166]. PAI-2 plays an important role in inflammation through the regulation of T-helper-cell activation [167]. The protein is expressed by activated macrophages but also endothelial cells and placental trophoblasts [166]. With data indicating an association between reduced PAI-2 mRNA and protein levels, increased Th1 activation, placental insufficiency, and endothelial dysfunction in PE, PAI-2 has been proposed as a potential contributor to pathogenesis in pregnancy. [52,167,168,169]. The serpin can also act as a holdase, a type of chaperone protein that can passively bind to prefibrillar protein species, preventing them from further aggregation [166,170,171]. PAI-2 was recently reported to inhibit Aβ aggregation and to block Aβ-induced cytotoxicity in vitro [136]. Contrary to PZP, PAI-2 co-localized with sPE-associated Aβ extracellular deposits in the placenta. Intriguingly, PAI-2 also co-localized with Aβ within syncytiotrophoblast cells, suggesting that PAI-2’s cytoprotective ability may occur through the stabilization of small conformational aggregates in the intracellular compartment. However, although the internalization of Aβ oligomers and fibrils by neurons and microglia is well established, the internalization of misfolded proteins by placental cells has not been shown, nor do we know the direct effect of Aβ on placental cell survival and function [172,173]. Another possibility is that intracellular cleavage of APP into Aβ results in the direct binding of PAI-2 to Aβ inside the cell, effectively sequestering the protein and reducing the amount of soluble detectable protein.

**Figure 4:**
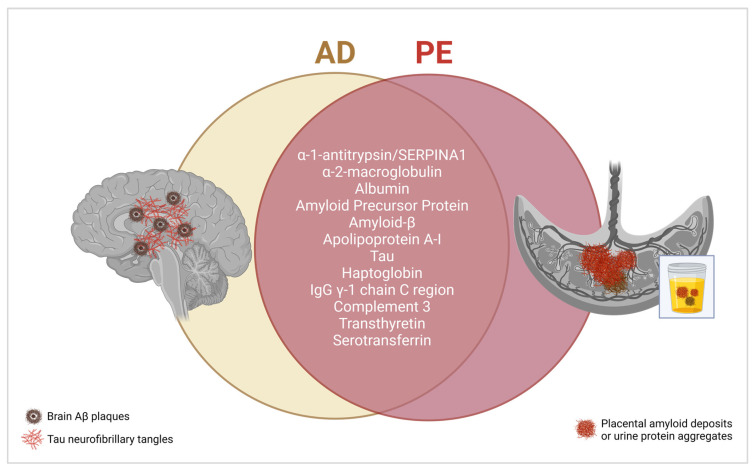
Shared proteins are found in AD-associated brain amyloid plaques and PE-associated placental and urine protein aggregates. Created with BioRender.com, accessed on 20 January 2024.

Interestingly, in most proteinopathies, though a primary amyloidogenic protein is identified, co-aggregation of multiple proteins is common. For example, in AD, hundreds of proteins associate with Aβ in brain amyloid plaques and in body fluids [174,175]. A comprehensive list of these proteins is available in a recent review by Rahman et al. [174]. We can note that there is considerable overlap between the proteins that interact with Aβ in AD and proteins that have been detected in PE placental and urine aggregates (Figure 4). Many of these proteins play a role in stabilizing different Aβ conformations, thus acting as chaperones. TTR is a good example of this phenomenon. Soluble TTR can bind to Aβ oligomers, effectively sequestering the unstable molecules and preventing their fibrillation [176,177]. This effect is highly apparent in the CSF and serum, where decreased TTR levels correlate with higher Aβ levels [178,179]. Recently, Lederer et al. reported an increase in CSF Aβ peptide levels in patients with HELLP syndrome; however, an inverse correlation with TTR levels is yet to be determined [180]. Since proteins like TTR are also prone to misfolding in PE, a loss of their chaperone function could explain the accumulation of Aβ in the serum, as observed by Cheng et al. [15].

### 5.3. Failure in Clearance Mechanisms: Defective Autophagolysosomal Processing

The third level at which disruptions in proteostasis mechanisms can occur is after all attempts at slowing the production of misfolded proteins and enhancing folding mechanisms have failed. At that time, misfolded proteins that exceed the UPR’s corrective capacity are targeted for degradation. Several studies and reviews have examined defects in autophagy as a potential root cause of protein aggregate accumulation in PE [14,122,134,181,182,183,184]. Autophagy is one of two processes through which cells clear defective proteinssuch as aggregated proteins, and, damaged intracellular components like organelles [63]. This process is mediated by a degradative enzyme in the lysosomes after the organelle fuses with autophagosomes carrying damaged cellular components (macroautophagy) or after the direct intake of individual unfolded proteins that were preemptively tagged (by ubiquitination) for degradation by chaperones (microautophagy) [185]. The second mechanism through which intracellular proteolysis occurs is through the ubiquitin–proteasome pathway. Upon ubiquitination, proteins may diverge to the proteasome, a large protein complex that can hydrolyze and degrade proteins [186]. Reduced efficiency of the autophagy machinery or dysfunction of the ubiquitin–proteasome system (UPS) have been extensively linked to the physiological accumulation of misfolded proteins during aging, as well as their pathological aggregation in AD [62,63,186]. Thus, assuming that defects in autophagy or the UPS can also underlie the accumulation of misfolded proteins in PE, especially in the context of sustained ER stress and UPR hyperactivation, is not a farfetched theory.

Nakashima et al. postulated a decade ago that autophagy induction is critical to protecting placental trophoblast cells from hypoxia-induced disruptions. They showed that relative to autophagy-competent cells, autophagy-deficient trophoblast cells exhibited poor invasion and decreased ability to induce vascular remodeling in response to hypoxia, suggesting that defects in autophagy may contribute to poor vascular remodeling [184,187]. Additional work by the same authors later showed decreased expression of lysosome-associated proteins LAMP1, LAMP2, CTSD, and TFEB; the regulatory protein that induces their expression is reduced in PE placentas [14]. On the other hand, SQSTM1/p62, a protein that mediates the insertion of ubiquitinated proteins to the autophagosome and is normally degraded when autophagy pathways are functional, was increased [14]. Finally, the authors showed that in Atg7^-/-^ mice, a lack of Atg7, a key effector in autophagosome biogenesis, resulted in the accumulation of aggregated proteins in the placental junctional zone [14].

Cheng et al. showed that both the blockade of autophagosome–lysosome fusion with chloroquine and that of proteasome activity via MG132 treatment cause the accumulation of TTR aggregates in PHTs [134,188]. Moreover, there was an irregular placental accumulation of ubiquitinated proteins in the setting of EOPE-associated ER stress and UPR. These results, while preliminary, support that the impairment of autophagy, but also of ubiquitination-mediated protein degradation processes, may play a role in PE-associated protein aggregation [134]. Evidence confirming the downregulation of autophagy in PE continues to accrue as Weel et al. described decreased LC3-II and beclin-1 mRNA and protein expression in the PE placenta [189]. Both LC3-II and beclin-1 are key markers of autophagosome formation and their expression correlates with the activation of the autophagic machinery [189,190]. On the other hand, there is a large body of literature showing that autophagy mechanisms are heightened in PE and not decreased [190,191,192,193,194]. The results reported by Weel et al. are in direct contradiction with early reports by Oh and co-authors of increased placental LC3-II expression in PE [190]. A more recent study by Oh et al. reported that in vitro inhibition of autophagy increases extravillous trophoblast cell invasion, in contrast with results from Nakashima et al. [184,195]. The nuances of these contradictory findings have been reviewed at length [182,183,196]. To explain these discrepancies, it has been proposed that early stages of the autophagic process, such as the formation of the autophagosome, may remain intact while later stages, such as fusion with the lysosome or autophagolysosomal activity, are impaired [14].

Ultimately, the three mechanisms we reviewed act as a coordinated system to maintain proteostasis. Failure at any or all three of these regulatory levels has the potential to drive the pathogenic accumulation of misfolded proteins. Even cellular responses, such as the UPR, which are meant to have protective effects, can result in a double whammy by exacerbating the production of misfolded proteins. While more research is needed to clarify the interaction between the different molecular pathways at play, having a hint that there is a dysfunction of these processes expands the strategies that can be used to detect and combat PE-associated proteostasis dysregulation.

## 6. New Prediction, Diagnostic, and Treatment Approaches Based on PE-Associated Protein Misfolding

### 6.1. Early Prediction and Diagnosis

To date, accurate and timely PE diagnosis continues to pose a major challenge in clinical practice. The recent exclusion of proteinuria as a compulsory diagnostic criterion was favorable for the detection of non-proteinuric PE cases but left the presence of elevated blood pressure as the only sign that can be used to initiate further [19,197]. Based on current clinical diagnostic guidelines, the syndrome can only be detected when overt symptoms are already present. To prevent adverse maternal and fetal outcomes, early prediction based on molecular pathophysiologic markers and diagnosis before symptom onset is critical. Beyond the assessment of existing risk factors, such as chronic hypertension, advanced maternal age, race, family history, or past medical history, that can give us a hint of pregnancies that have a higher risk of PE occurrence, accurate prediction of PE was not possible until the advent of the sFlt-1/PlGF ratio as an effective clinical decision-making tool [43,198]. With the knowledge that PE is a protein conformational disorder, misfolded proteins in the urine or plasma are additional biomarkers that can be targeted to expand our diagnostic toolset.

Methods that have been developed in AD can inform us of the potential of misfolded protein detection to accurately predict and diagnose proteinopathies. For example, plasma and CSF assays assessing Aβ42/Aβ40 and tau/P-tau levels among other biomarkers are highly successful at identifying the presence of AD pathology prior to the threshold for amyloid positivity on PET scans and the onset of cognitive decline and at predicting disease trajectory [199,200,201]. The biomarker assays perform even better when combined with genetic risk score [202]. APOE-ε4, one of three alleles of the APOE gene, is well-established as the greatest genetic risk factor for AD. Adding APOE-ε4 status alone to prediction models increases AUC and, thus, predictive value [202,203]. A role for APOE-ε4 has previously been proposed in PE risk, based on the protein’s lipid modulatory functions. But an association between the allele and PE-associated protein misfolding features has not been investigated. Identifying clear genetic potentiators of PE risk, specifically genes, like APOE, which are associated with protein misfolding in other proteinopathies, can improve our current ability to recognize pregnancies that are most likely to be impacted by PE.

A novel blood-based assay that can detect the presence of misfolded Aβ, TTR, or P-tau in the maternal circulation has been proposed by Cheng et al. [15]. This method uses an autophagy-deficient trophoblast cell model to establish whether misfolded proteins are present in serum. The exposure of these specific autophagy-deficient human trophoblasts (ADTs) to serum from patients with PE, AD, and mild cognitive impairment leads to the intracellular accumulation of protein aggregates made of TTR and Aβ in PE cases, with the addition of α-synuclein in AD and MCI cases [15]. Using this detection medium would be particularly useful in non-proteinuric or pre-proteinuric patients. However, the system requires highly technical expertise in cell culture (including access to a biosafety cabinet, ultra-low temperature freezers for storage, and tissue culture incubators) and the use of a confocal microscope [15]. This may be prohibitive in low-resource settings where the burden of PE-associated morbidity and mortality is highest but access to these high-technology tools is not readily available [16]. More studies are needed to understand the feasibility and performance of this method in a clinical context.

The Congo red dot (CRD) test represents a non-invasive and low-cost alternative to other methods of assessing protein misfolding load associated with PE. The clinical applicability of the CRD test, which is based on the detection of urine congophilia, as established by our lab, has been examined in fourteen studies, including one randomized control trial and one meta-analysis ([90,91,204,205,206,207,208,209,210,211,212,213,214,215]). In one study, our lab evaluated the performance of the CRD test at measuring misfolded protein load in urine and serum against that of Thioflavin-T (ThT)-enhanced fluorescence, a benzothiazole dye that preferentially binds to amyloid fibrils [214]. While both urine and serum ThT-enhanced fluorescence could efficiently predict PE, urine %CRR, as determined by CRD testing, had a high AUC and, thus, the ability to identify women who had a medically indicated delivery for PE (MIDPE) [214]. The CRD test, which was initially optimized for that purpose and for its utility in ruling out PE in women presenting with symptoms in a US population, was also tested in a variety of other clinical contexts and diverse populations as a point-of-care test [12,212,213]. Nagarajappa et al. showed the utility of the test in differentiating preeclamptic from normotensive pregnancies in an Indian cohort of pregnant women [91]. A second study in India reported similar conclusions, in addition to the ability to predict PE and discriminate the disorder from other hypertensive disorders of pregnancy [207]. Rodriguez Chavez and co-authors determined that the CRD test is a highly selective screening test for the detection of misfolded proteins in the urine, after testing the method on 50 pregnant women in the state of Mexico. [215] The CRD test method also proved effective at screening PE in a Chinese cohort of pregnant women presenting in late pregnancy (>28 weeks); although, the authors used a modified technique to apply the urine–Congo red mix onto the nitrocellulose membrane [211].

On the other hand, a few studies report low test performance. Results from Sammar et al. showed that the CRD test can diagnose PE with high accuracy in women presenting with symptoms; however, that the test is less effective at predicting PE in the first trimester of pregnancy when used alone [208]. The predictive value of the test was improved when %CRR was used in combination with other markers of high disease risk, such as previous PE diagnosis, black race, body mass index, and mean arterial pressure [208]. In addition, the CRD test was not efficient at predicting PE in a term asymptomatic cohort (UK) and was determined to have low sensitivity at predicting impending MIDPE (within 28 days of initial assessment) in a randomized control trial, as well as low sensitivity at predicting adverse maternal and neonatal outcomes in women presenting with symptoms of suspected PE [205,206,210]. Finally, in a meta-analysis that included five studies, Khaliq et al. concluded that the test is ineffective based on forest plot analysis [204]. Of note, the five studies compared in the meta-analysis comprised highly heterogeneous patient populations. Additionally, factors such as population-specific disease prevalence, differing scoring and cutoff criteria, disparate clinical definitions of PE, clinical contexts divergent from the initial intended purpose, and the use of different embodiments of the test (point of care device, nitrocellulose membrane, differing paper types) are all important variables that influence test sensitivity and confound the interpretation of the results.

Finally, the test specificity has also been challenged in the setting of pre-existing or co-existing kidney disease [90,209]. In both studies, %CRR was comparable between women with PE and women with CKD [90,209]. Moreover, Younis et al. showed that the burden of urine congophilia was similar between women with PE and women with pregnancy-related acute kidney injury from causes other than PE [209]. Kidney injury is a key component of PE symptomatic manifestations [54]. As previously addressed, pre-existing kidney disease is also a precipitating risk factor for PE [55,56]. Since the pathophysiology of PE and kidney disease/injury are so intertwined, it is possible that the use of a urinary biomarker for disease detection leads to the indiscriminate recognition of either. In clinical practice, clinical management of pregnant women with pre-existing kidney disease requires specialized care that may not require the need for point-of-care diagnostic testing. Additionally, given the progressive nature of the PE syndrome, it may not be possible to fully rule out the possibility that a hypertensive phenotype would not have developed sometime in the future. More research is necessary to determine the utility of the CRD test in pregnant women with suspected concomitant disease.

### 6.2. Treatment

Other than the emergent delivery of the fetus and placenta, which comes with its own set of iatrogenic complications when conducted preterm, there is no effective therapy for PE. While we are still decades away from fully understanding the mechanisms at play in the development of protein misfolding in PE, the knowledge that disturbances in proteostasis occur offers new molecular pathways that can be targeted for treatment. Novel strategies that can be employed to reduce the accumulation of misfolded proteins and prevent their toxic effects include the inhibition of ER stress, stabilization of chaperone function, or restoration of autophagic balance. For example, the stabilization of TTR’s tetrameric structure is currently being explored as a therapeutic mechanism for the treatment of FAP and AD. Dissociation of the tetramer into monomers potentiates TTR’s aggregation into fibrils in FAP and compromises TTR’s ability to sequester unstable Aβ conformations in AD [129,132,216,217]. A handful of small-molecule pharmacological chaperones that can stabilize TTR have shown promise in in vitro and in vivo studies (for AD) and clinical trials (FAP); however, none of these compounds would be safe for use in pregnancy [132,217,218,219].

ER stress modulators, autophagy-enhancing drugs, gene therapy, and anti-amyloid immunotherapy are also being examined as therapeutic approaches in the treatment of AD [146,220,221,222]. In recent years, sildenafil, a known inducer of autophagy, has been associated with the alleviation of cognitive impairment in pre-clinical models and decreased incidence of AD in human studies and, thus, has been proposed as a candidate drug that could be repurposed for AD treatment [223,224]. The potential of sildenafil use for the treatment of AD has not yet been verified as evidence from a recent study does not support previous claims [225]. In PE, sildenafil, which is safe to use in pregnancy, has also been investigated as a potential treatment option based on its vasodilating function and has shown modest results in improving uteroplacental blood flow and fetal growth in clinical studies [226,227,228]. The drug has also been shown to attenuate oxidative stress and protect against kidney injury in animal models [229]. Whether the potential benefits of sildenafil treatment are linked to the induction of autophagy and subsequent decrease in misfolded protein burden is unknown. However, what is known is that the administration of sildenafil in the context of severe early-onset FGR may be harmful to the neonate. The STRIDER Canada trial, a national multisite double-blind, placebo-controlled randomized controlled trial aimed at determining the efficacy and safety of sildenafil, was discontinued due to an increased risk of neonatal pulmonary hypertension and a non-significant trend toward increased neonatal mortality [230,231]. Safety remains the foremost consideration in the development of any therapeutic method aimed at targeting protein misfolding mechanisms.

Importantly, after decades of pre-clinical development and many clinical failures, immunotherapy approaches for the treatment of AD have finally shown success in phase-three clinical trials [232]. Three monoclonal antibodies (aducanumab, lecanemab, and donanemab), which target aggregated forms of Aβ, have been shown to effectively reduce brain amyloid deposits and slow cognitive and functional decline in the early stages of AD [233,234,235]. Each of the antibodies has a preferential binding affinity toward specific conformations and only has a low affinity toward Aβ monomers, which may explain differences in their efficacy and side effect profile [236]. Nevertheless, because patients with PE and AD have overlapping Aβ conformers, there may be a rationale for exploring misfolded proteins as targets for immunotherapy for PE [94].

Though the use of any of these approaches to treat PE is still an idea in its infancy, close attention should be paid to their potential. Given the multiple pathological processes at play, in PE, there is no guarantee that strategies aimed at decreasing protein misfolding load will be sufficient enough to alleviate PE-onset or severity. Additionally, approaches aimed at targeting a single type of misfolded protein may prove to be inefficient due to the presence of multiple proteins in PE aggregates. More research is needed to identify ideal targets for the alleviation of PE-associated protein misfolding pathology.

## 7. Long-Term Implications of Pregnancy-Related Protein Misfolding Disease on Maternal and Fetal Health

As the field progresses in its understanding of PE-associated disease processes, the more complex the etiology of the disorder seems to become. Current evidence seems to point to a multisystem multifactorial pathological process, which extends beyond placental dysfunction. Gaining a better understanding of protein misfolding mechanisms and how they may contribute to systemic dysfunction in PE is crucial to advancing our ability to care for mothers and their babies during pregnancy. On the other hand, it also gives us a window into their future health. It is well-established that having PE during pregnancy greatly increases the risk of cardiovascular disease later in life for both mothers and their offspring [237,238,239,240]. Accumulating epidemiological reports also suggest a link between hypertensive disease during pregnancy and long-term risk of vascular dementia and AD [241,242]. Strikingly, women also have a two-fold greater risk of developing AD compared to men [243]. While lifetime hormonal fluctuations, parity, lingering neuroinflammation, and neurovascular damage have been proposed as contributors to this sex-specific effect, the onset of protein misfolding during PE establishes another link between PE and AD [244,245,246,247]. Perhaps the accumulation of misfolded proteins during pregnancy accelerates the development of similar pathology in the brain, especially if Aβ and tau pathology are involved. The link between protein aggregation in PE and long-term risk has not been investigated in vivo and constitutes another area where research could be expanded.

While the discovery of significant protein misfolding pathology in PE opens many new avenues for investigation, diagnosis, and treatment, many questions are left to be answered. The central questions that persist and still require attention include whether protein aggregation instigates PE or is a consequence of it; the reasons behind the selective detection of certain proteins in specific tissues (serum vs. placenta vs. urine); and the systemic factors that may affect the accumulation and deposition of misfolded proteins in pregnancy. The possibility that proteostasis disruption might occur systemically rather than being confined at the placental level carries significant implications for both immediate and long-term maternal and fetal health. Consequently, careful consideration of how the pregnancy phenotype might play into the development of other proteostasis disorders later in life is warranted.

## Figures and Tables

**Table 1:** List of amyloidogenic proteins identified in PE across eight major publications.

Protein	Urine	Serum	Placenta
**α-1-antitrypsin (SERPINA1)** ^†^	Buhimschi [12,13] + Sergeeva [93] +	Cheng [15] **−**	Buhimschi [13] +
**α-1-antitrypsin/FVFLM fragment** ^†^	Sergeeva [93] +		
α-2-macroglobulin	Sergeeva [93] +		
Albumin	Buhimschi [12,13] + Sergeeva [93] +		
**Amyloid-β** ^†^	Valtanen [94] + Buhimschi [12] +	Cheng [15] +	
Apolipoprotein A–I	Sergeeva [93] +		
**Amyloid precursor protein (APP)** ^†^	Buhimschi [12] + Sergeeva [93] **−**		Buhimschi [12] +
α-synuclein		Cheng [15] **−**	
Ceruloplasmin	Buhimschi [12] + Sergeeva [93] +		
**Cis p-tau** ^†^		Jash [98] +	Jash [98] +
Complement 3	Sergeeva [93] +		
Haptoglobin	Sergeeva [93] +		
IgG γ-1 chain C region	Sergeeva [93] +		
IgG k-free light chain	Buhimschi [12] +		
Interferon-inducible protein 6	Buhimschi [12] +		
Serotransferrin	Sergeeva [93] +		
**Transthyretin** ^†^	Sergeeva [93] +	Cheng [15] + Kalkunte [11] +	Tong [99] +
Trypstatin	Sergeeva [93] +		

^†^ Bolded proteins will be discussed in more detail. A positive (+) sign indicates references that identified the presence of the indicated protein in the urine, serum, or placenta. A negative (−) sign indicates references that did not identify the presence of the indicated protein in the urine, serum, or placenta.
